# Effects of physical activity on mobile phone addiction among university students: the mediating roles of self-control and resilience

**DOI:** 10.3389/fpsyg.2025.1503607

**Published:** 2025-03-31

**Authors:** Fang Wang

**Affiliations:** School of Sports Training, Chengdu Sport University, Chengdu, China

**Keywords:** physical activity, self-control, resilience, mobile phone addiction, college students

## Abstract

**Background:**

In recent years, mobile phone addiction (MPA) has emerged as a significant public health concern, particularly among university students. Physical activity (PA) is believed to exert a beneficial influence on MPA within this demographic. However, the extent to which this influence is moderated by other factors remains uncertain. Evidence points to self-control and resilience as potential mediators that may partially account for the positive impact of PA on MPA. Accordingly, this study seeks to investigate the effect of PA on MPA and the mediating role of self-control and resilience in this relationship through a chain-mediated model.

**Methods:**

The study involved 413 Chinese university students (208 males, mean age 20.59 ± 1.17 years), who completed the PA Rating Scale (PARS-3), the Connor-Davidson Resilience Scale (CD-RISC), the Self-Control Scale (SCS), the Mobile Phone Addiction Index (MPAI), along with other psychosocial assessments. Pearson’s correlation was employed to analyze the relationships between variables, while mediation models were examined using SPSS PROCESS and bootstrapped regression analysis.

**Results:**

PA demonstrated a significant negative correlation with mobile phone addiction behaviors (β = −0.22, *p* < 0.01). Self-control and resilience moderated the relationship between PA and mobile phone addiction. Notably, PA influenced mobile phone addiction through the chain-mediated effects of self-control and resilience.

**Conclusion:**

College students can alleviate MPA issues by consistently engaging in healthy PA, which is essential for enhancing self-control and bolstering resilience.

## 1 Introduction

The advent of smartphones is akin to a double-edged sword, offering convenience to people’s studies, work, and daily lives, while simultaneously fostering the issue of mobile phone addiction. Mobile phone addiction (MPA), also referred to as problematic mobile phone use, is a detrimental behavior linked to excessive smartphone usage. This phenomenon is particularly evident in individuals’ profound desire and reliance on mobile devices, resulting in excessive engagement and usage of these technologies (Billieux et al., 2015; Zou et al., 2017). According to the World Health Organization, MPA has emerged as a significant public health concern, particularly among adolescents (Haug et al., 2015). Some experts predict that it will become one of the most widespread forms of technology addiction in the 21st century (Busch and McCarthy, 2021). The report of the China Internet Information Centre (CINIC) realistically shows that mainland China’s Internet users reached 854 million in 2019; 99.1% of them accessed the Internet through mobile phones (China Internet Network Information Center, 2019). Among them, those aged 20–29 accounted for 24.6% of the total proportion, while the college student group applied mobile phones for more than 5 h a day on average, and about 79% of students applied mobile phones in class (MyCOS Research Institute, 2018). Recent studies indicate that adolescents and young adults constitute a significant demographic in mobile phone usage, exhibiting a notably high prevalence of MPA, especially among college students (Li et al., 2020; Lissak, 2018; Sohn et al., 2019). The detrimental effects of MPA on the college population are profound, potentially resulting in physical ailments such as dizziness, headaches, stiffness, limb numbness, reduced spinal strength, visual disturbances, and even loss of vision. Furthermore, it can give rise to mental health issues, including procrastination, depression, anxiety, fear, and insomnia (Elhai et al., 2019; Thomée, 2018). As a result, MPA is increasingly being recognized as a public health problem and there is a need to identify its causative factors and develop coping strategies to prevent and mitigate this disturbing behavior.

Within the field of research on how to reduce and mitigate the phenomenon of MPA, the academic community has taken note of physical activity as an effective modality (Chen et al., 2022; Yang et al., 2021). PA serves as a means for individuals to achieve fitness and health, engage in leisure and enjoyment, and bolster both physical and mental well-being. It enhances physical fitness, improves overall health, and sustains the body’s capabilities (WHO, 2007, 2010). The World Health Organization (WHO) advises a minimum of 150 min per week of moderate-to-vigorous intensity PA to prevent chronic diseases. Additionally, it recommends engaging in 75 min of high-intensity exercise weekly, as regular PA offers substantial benefits for both physical and mental health (WHO, 2015). Existing studies have demonstrated that exercise serves as an effective intervention for the treatment of MPA, with longer intervention durations yielding more favorable outcomes (Liu et al., 2019). Systematic PA can significantly alleviate smartphone addiction (Li et al., 2023). Recent research indicates that PA acts as an effective negative predictor of MPA, with self-control mediating the relationship between the two (Guo et al., 2022). It is suggested that regular PA is a viable strategy for preventing MPA, and there is moderate evidence supporting its effectiveness in reducing addictive behaviors among university students (Pirwani and Szabo, 2024). The findings of the studies highlight the potential benefits of PA, which may serve as a crucial protective mechanism against MPA. Therefore, given that previous research has focused on the link between PA and MPA. In the current setting, there is more room for potential mediating mechanisms (i.e., how PA affects MPA) to be explored. In addition, while there has been external research on the relationship between individual traits such as anxiety, social impairment, and loneliness and MPA, there has been relatively little research on self-control and mental toughness on MPA. To fill these gaps, the present study constructed a chain mediation model to examine the mediating roles of self-control and mental toughness between PA and MPA. In contrast to previous studies, the purpose of this study was to further expand the mechanistic exploration of the relationship between PA and MPA. To add to the research gap regarding self-control and resilience as relevant variables in this area. To further contribute to the academic understanding of the mechanisms by which PA influences MPA in the university student population, thereby informing higher education professionals to optimize the mental health and MPA phenomenon in university students.

### 1.1 The mediating role of self-control

Self-Control Theory (SDT) states that self-control is negatively associated with addictive behaviors, suggesting that PA might reduce MPA by enhancing self-regulation. Self-control refers to an individual’s capacity to regulate their own behavior and align personal values with societal expectations (Kopp, 1982). It facilitates or inhibits specific behaviors, such as resisting impulses, withstanding temptations, and delaying gratification (Tangney et al., 2004). The self-control model likens self-control to a muscle, suggesting that, much like a muscle can be strengthened through regular exercise, self-control can be enhanced through practice and effort (Yang et al., 2019). Studies have indicated that self-control plays a crucial role in MPA behavior, significantly serving as a negative predictor of such behavior (Pan et al., 2023). Typically, MPA is associated with impulsivity, which positively predicts MPA, while self-control effectively curtails impulsive tendencies (Burnell and Kuther, 2016).

Recent studies have confirmed that PA can improve college students’ self-regulation by increasing self-control, which in turn indirectly improves the frequency of mobile phone use among college students through self-control, among other things. Additionally, research has shown that self-control can mediate the relationship between bullying victimization and MPA among college students, with PA moderating this relationship (Liu et al., 2024; Zeng et al., 2022). Self-control may significantly influence the effect of PA on MPA, as research has shown that participation in PA among adolescents enhances self-control, thereby reducing dependence on mobile phones (Zhang et al., 2022). Therefore, this study hypothesized that self-control mediates the relationship between PA and MPA.

### 1.2 The mediating role of resilience

Resilience is the capacity of an individual to adapt effectively in the face of challenges, trauma, adversity, significant stress, or even tragedy (Cousins and Fernández, 2019). It signifies a state of resilience that enables a person to regain normalcy after encountering trauma, accidents, tragedy, or illness, a quality vital for both physical and mental well-being (Babić et al., 2020). Research on resilience indicates that PA promotes coping abilities, reducing reliance on mobile phones for emotional support. Research has shown that PA effectively enhances the resilience of college students, further improving their problem-solving abilities, self-confidence, and emotional regulation (Deng et al., 2023; Peng and Liu, 2010). In contrast, the self-attrition model of self-regulatory capacity posits that individuals who struggle to adapt to social challenges may experience a decline in self-control while using mobile phones.

Adaptive Capacity Theory proposes that PA improves psychological resilience, which helps mitigate addiction risks. At the same time, mental toughness is strongly associated with PA. PA is an effective way to improve resilience. Moreover, it has been demonstrated that PA can mitigate the risk of MPA by bolstering resilience and reducing perceived stress (Zhao et al., 2022). Consequently, it can be inferred that when college students experience a decline in self-control, their resilience is similarly impacted, fostering an increased dependence on mobile phones that may lead to addiction (Ma et al., 2022a). Therefore, this study hypothesized that resilience is the mediating variable between PA and MPA.

### 1.3 Chain mediation of self-control and resilience

Evidence suggests that low self-control and poor resilience to setbacks may be important factors in college students’ MPA (Ma et al., 2022b; Niu, 2023), and self-control and resilience may play a chain mediating role in this process, a relationship based on previous research (Wu et al., 2024). However, the testing of the model remains subject to further validation to gain the fullest understanding. Exploration of this relationship may lead to a deeper understanding of the interaction between PA and MPA in college students, with existing theories providing insights into the link between self-control and resilience and MPA. For example, self-control theory suggests that low self-control may lead to individuals who are inattentive and may take extreme measures in the face of stress, including addictive behaviors. Some studies indicate a significant positive correlation between self-control and resilience (Liang et al., 2022). In addition, compensatory network use theory further hypothesis that poor resilience may further contribute to an individual’s MPA when they experience psychological stress, depression, anxiety, etc., resulting in unmet needs related to social interaction, recreation, and identity. Therefore, it is hypothesized that an individual with low self-control and poor resilience will have an increased tendency to develop MPA. Furthermore, the PA behaviors of college students may enhance individual self-control and improve their capacity to adapt to setbacks and manage negative emotions. Conversely, low levels of participation in PA, along with diminished self-control and resilience, may exacerbate MPA, thereby impacting physical and mental health (Schuch et al., 2020). Based on this, the positive effects of PA on MPA may stem from increased self-control and resilience. Therefore, this study hypothesized that self-control and resilience play a role in the relationship between PA and MPA.

### 1.4 Research hypotheses

**Figure 1:**
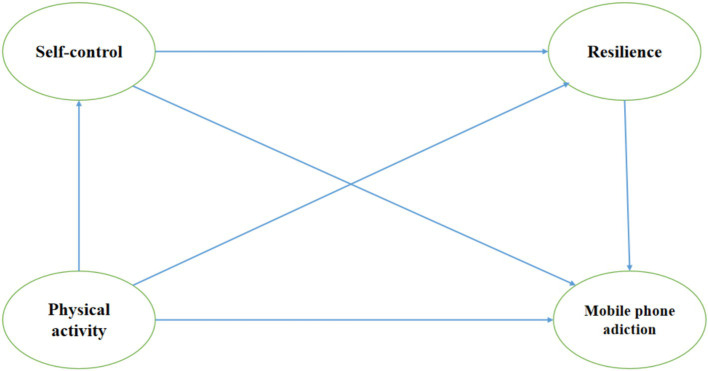
Research model.

The purpose of this study was to explore the mediating roles of self-control and resilience between physical activity and MPA among Chinese college students, aiming to broaden and consolidate our understanding of the mechanisms associated with physical activity and MPA. Based on previous research, this study provides a preliminary exploration of the interrelationships between physical activity, self-control, resilience, and MPA. Notably, previous studies have focused on the pairwise relationships between these factors (Wu et al., 2024; Zhang et al., 2022), and there is a gap in how self-control and resilience influence the correlations between physical activity and MPA and the pathways of interconnections. Therefore, this study examines the effects of PA on MPA among college students based on self-determination theory, substitution hypothesis, stress dissipation hypothesis, and adaptive capacity theory. In addition, this study aims to explore the mediating roles of self-control and resilience to consolidate and expand the understanding of the mechanisms underlying the relationship between PA and MPA. Based on the above theories and discussions, the following hypotheses and hypothesized models were proposed in this study (e.g., Figure 1).

*H1:* Physical activity negatively predicts mobile phone addiction.

*H2:* Self-control mediates the relationship between physical activity and mobile phone addiction.

*H3:* Resilience mediates the relationship between physical activity and mobile phone addiction.

*H4:* Self-control and resilience function the relationship between physical activity and mobile phone addiction.

## 2 Methods

### 2.1 Participants

According to calculations of effect size using G*Power software, the minimum sample size required is 300 (Schoemann et al., 2017). Consequently, this study employed a convenience sampling method to administer an open-ended questionnaire survey to 450 students at an undergraduate institution in Chengdu, Sichuan Province, China, through both offline face-to-face interactions in the classroom and online distribution of the questionnaire. We hereby certify that this study was conducted in accordance with the 1964 Declaration of Helsinki and its subsequent amendments. Furthermore, we confirm that all methodologies employed in this study received approval from the Ethics Committee, and we affirm that informed consent was obtained from all participants (approval number: CTYXLL2024004).

The questionnaires were clearly and objectively explained to the participants prior to distribution, and they were completed independently with the subjects’ consent. At the end of the whole survey, 37 invalid questionnaires or questionnaires that did not meet the requirements (including blank questionnaires and omitted questionnaires) were excluded, and there were 413 valid questionnaires, with a validity rate of 91.78%. Among the participants, there were 208 males and 205 females, with an average age of 20.59 ± 1.17 years. This group included 86 freshmen, 118 sophomores, 146 juniors, and 63 seniors or above, 189 in Physical Education, and 224 in Sports Training. Please refer to Table 1 for further details regarding the subjects.

**Table 1:** Descriptive statistics of the participant population (*n* = 413).

Project	Category	*N*	Percentage
Genders	Male	208	50.36%
	Female	205	49.64%
Age	19	90	21.79%
	20	106	25.67%
	21	126	30.51%
	22	65	15.74%
	23	26	6.30%
Grades	Freshman	86	20.82%
	Sophomore	118	28.57%
	Junior	146	35.35%
	Senior	45	10.90%
	Fourth year or above	18	4.36%
Major	Physical education	189	46.76%
	Sports training	224	54.24%

### 2.2 Measurement tools

#### 2.2.1 Physical activity rating scale (PARS-3)

The Physical Activity Rating Scale-3 (PARS-3) utilized in this study was revised by the Chinese scholar Liang Deqing, based on the original version introduced by the Japanese scholar Kenji Hashimoto in 1990 (Liang, 1994). This scale was employed to assess participants’ levels of physical activity in terms of intensity, frequency, and duration. The indicators were categorized into five levels and rated on a scale from 1 to 5. The total score for physical activity is calculated as follows: Total score = intensity of exercise × (duration of exercise − 1) × frequency of exercise, with a range from 0 to 100 points. A higher score indicates a greater level of physical activity. Specifically, a score of ≤19 denotes a small amount of exercise; a score between 20 and 42 signifies a moderate amount; and a score of ≥43 indicates a large amount of exercise. In this study, the Cronbach’s α coefficient for the scale was found to be 0.813.

#### 2.2.2 Resilience scale (CD-RISC)

In order to assess the psychological resilience of Chinese university students and to avoid problems that may occur with scales in different populations and cultural contexts, the Chinese Revised Connor-Davidson Resilience Inventory Scale (CDRISC) was used in the present study and has been confirmed by previous research to have valid structural validity and to be suitable for use in the conduct of this study (Yu and Zhang, 2007). The scale comprises 25 items and encompasses three primary dimensions: resilience, strength, and optimism. Participants rated each item on a 5-point Likert scale, ranging from 1 (almost never) to 5 (almost always). Higher scores reflect greater resilience. Measurement model fit results were *X*^2^/df = 1.022, GFI = 0.936, CFI = 0.999, TLI = 0.999, RMSEA = 0.008. The overall Cronbach’s α coefficient for the CD-RISC was 0.936.

#### 2.2.3 Self-control scale

This study utilized a revised version of the Self-Control Scale (SCS) developed by Morean et al. in 2014, which resulted in a streamlined seven-item version encompassing two dimensions (Morean et al., 2014). In comparison to the original version from 2004, this revised scale significantly reduces response fatigue and bias, and it has been translated into multiple languages, demonstrating cross-cultural consistency in its reliability and validity. Measurement model fit results were *X*^2^/df = 1.516, GFI = 0.970, CFI =0.992, TLI = 0.990, RMSEA = 0.038, and SRMR = 0.028, so the scale has good construct validity. The SCS‘s Cronbach’s α coefficient was 0.925 overall.

#### 2.2.4 Mobile phone addiction (MPAI)

To evaluate MPA, researchers employed the MPA Index Inventory (MPAI) (Leung, 2008). This index consists of 17 items assessing four dimensions of MPA: lack of control over cravings, anxiety levels, disorientation and withdrawal, and decreased productivity. Survey respondents rated their experiences on a 5-point scale, ranging from 1 (never) to 5 (always). Previous studies have confirmed the reliability and validity of the MPA among Chinese adolescents and young adults. In the current study, the measure demonstrated strong internal consistency. The fitted results of the measurement model were *X*^2^/df = 0.865, GFI = 0.993, CFI = 0.92, TLI = 0.95, RMSEA = 0.0001, and SSRMR = 0.017. The MPAI Cronbach’s α coefficient was 0.924.

### 2.3 Data analysis methods

Analyses were conducted using the SPSS statistical package (version 26.0). Categorical variables were represented as frequencies and percentages. This study used gender-adjusted analysis of covariance (ANCOVA), independent samples, and rank sum tests and considered the outcomes of Kolmogorov and normal distribution tests to assess differences in PA, MPA, self-control, and resilience. For parametric or non-parametric tests that produced a *p*-value, effect sizes, specifically Cohen’s d or Pearson’s r, were calculated accordingly.

To examine the potential mediating role of self-control and resilience in the relationship between PA and MPA, the models were analyzed in this study using Model 6 from the SPSS Process 4.0 plug-in developed by Hayes (2013). In this model, PA serves as the independent variable (X), while self-control and resilience act as the mediating variables (M), and MPA is the dependent variable (Y). Considering the potential impact of age and gender on MPA, they were also considered as covariates in this study. This analysis enabled us to assess the mediating effects of self-control and resilience between PA and MPA. Consequently, the analysis was conducted using SPSS, and the mediation hypothesis was evaluated using a bias-corrected bootstrap method on a sample of 5,000, with 95% confidence intervals calculated. Significance was determined when the confidence interval did not encompass zero.

## 3 Results

### 3.1 Common method bias

Common methods bias was assessed using Harman’s one-factor method, alongside the KMO and Bartlett’s test, yielding a KMO value of 0.974 and a Bartlett’s statistic of 11961.872 (*df* = 741, *p* < 0.001), indicating that the data were appropriate for factor analysis. Consequently, an exploratory factor analysis of the items and each scale was conducted in this study. The results revealed that 20 factors had eigenvalues exceeding 1, with the first factor accounting for only 39.38% of the total variance, falling short of the 50% threshold. Thus, no common method bias was present in the data for this study.

### 3.2 Correlation analysis

According to the results presented in Table 2, PA exhibited a significant positive correlation with both self-control and resilience. Self-control also demonstrated a significant positive correlation with PA and resilience, while PA, self-control, resilience, and MPA showed significant negative correlations. In essence, these findings indicate interrelationships among the variables, suggesting that self-control and resilience may exert an influential role in the connection between PA and MPA, thereby establishing a foundation for further exploration of the mediating effects in subsequent analyses.

**Table 2:** Correlation analysis of all variables.

Variables	Mean (M)	Standard deviation (SD)	1	2	3	4
PA	31.9	29.537	1	–	–	–
Self-control	3.3175	0.93135	0.402^**^	1	–	–
Resilience	3.3768	0.92315	0.418^**^	0.452^**^	1	–
MPA	2.6949	0.93153	−0.447^**^	−0.432^**^	−0.408^**^	1

**p < 0.01.

### 3.3 Chain mediation effect test

**Figure 2:**
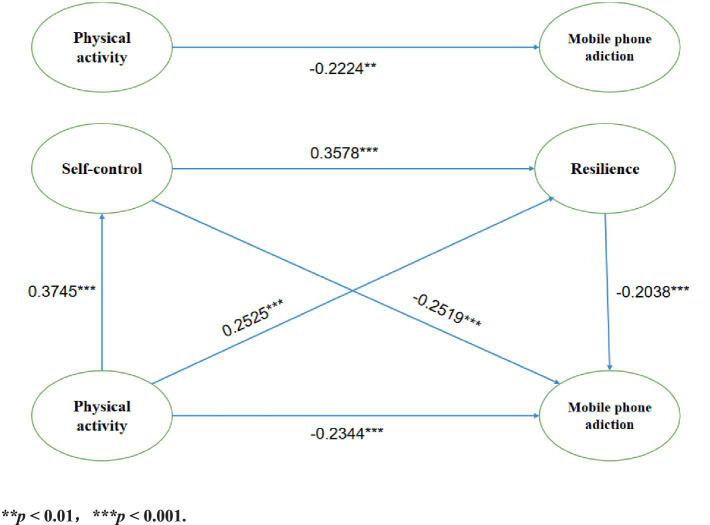
Regression analysis of the chained intermediation model. **p < 0.01, ***p < 0.001.

Based on Model 6 in the Process plug-in, a chained mediation model was established with PA as the independent variable, self-control and resilience as mediator variables, and MPA as the dependent variable. In this study, a replicate sample of 5,000 was utilized, with a default confidence interval of 95%. As indicated by the regression analyses in Table 3 and Figure 2, all three variables—PA (β = −0.2344, *p* < 0.001), self-control (β = −0.2519, *p* < 0.001), and resilience (β = −0.2038, *p* < 0.001) demonstrated significant negative predictive effects on MPA. Furthermore, PA was identified as a significant positive predictor of both self-control (β = 0.3745, *p* < 0.001) and resilience (β = 0.2525, *p* < 0.001). Additionally, self-control emerged as a significant positive predictor of resilience (β = 0.3578, *p* < 0.001). These findings suggest that self-control and resilience mediate the relationship between PA and MPA.

**Table 3:** Chained model regression analyses for PA, MPA, self-control and resilience.

Regression equation		Overall fit index			Regression coefficient				
Resulting variables	Predictor variables	*R*	*R* ^2^	*F*	β	*SE*	*t*	LLCI	ULCI
Self-control	PA	0.3745	0.1402	67.0354^***^	0.3745	0.434	8.1875^***^	0.2699	0.4405
Resilience	PA	0.5093	0.2594	71.8151^***^	0.2525	0.431	5.5084^***^	0.1527	0.3221
	Self-control	–	–	–	0.3578	0.0454	7.8065^***^	0.2654	0.444
MPA	PA	0.5362	0.2875	55.0205^***^	−0.2344	0.0443	−5.0246^***^	−0.3094	−0.1354
	Self-control	–	–	–	−0.2519	0.0483	−5.2214^***^	−0.3468	−0.1571
	Resilience	–	–	–	−0.2038	0.0489	−4.2028^***^	−0.3019	−0.1095

**p < 0.01, ***p < 0.001.

The overall path coefficients for the mediation analyses are presented in Table 4. In the total effects model, the direct predictive effect of PA on MPA was significantly negative (β = 0.2224, 95% CI [−0.3094, −0.1354]). When self-control and resilience were incorporated as mediating variables in a chained mediation model, the overall mediation effect was calculated at −0.1642, representing 42.47%. The 95% CI [−0.2171, −0.1155] does not include zero, thereby indicating that self-control and resilience function as chain mediators within this model. Secondly, by incorporating self-control and resilience into the relationship between PA and MPA, we identified four pathways through which PA influenced MPA: Path 1 represented the direct effect of PA on MPA (β = 0.2224, 95% CI [−0.3094, −0.1354]), accounting for 57.53% of the effect; Path 2 illustrated the mediating role of self-control between PA and MPA (β = −0.0895, 95% CI [−0.1325, −0.0511]), contributing 23.15% to the effect; Path 3 detailed the mediating role of resilience between PA and MPA (β = 0.0488, 95% CI [−0.0797, −0.0242]), which represented 12.62% of the effect; and Path 4 examined the combined mediating role of self-control and resilience in the chained mediation between PA and MPA (β = 0.0259, 95% CI [−0.0442, −0.0122]), accounting for 6.70%. The 95% confidence intervals for all four pathways did not include zero and did not exceed one, indicating that PA, self-control, and resilience can independently influence MPA. Furthermore, self-control and resilience can jointly function as chain mediators in the relationship between PA and MPA among college students, providing supporting evidence for the hypotheses.

**Table 4:** Chain mediation test for PA, MPA, self-control and resilience.

	Effect size	Boot SE	95% confidence interval		Proportion of relative effect
			Lower	Upper	
Total effect	−0.3866	0.0427	−0.4706	−0.3026	100.00%
PA → MPA	−0.2224	0.0443	−0.3094	−0.1354	57.53%
PA → Self-control → MPA	−0.0895	0.0201	−0.1325	−0.0511	23.15%
PA → Resilience → MPA	−0.0488	0.0144	−0.0797	−0.0242	12.62%
PA → Self-control → Resilience → MPA	−0.0259	0.0081	−0.0442	−0.0122	6.70%
Total intermediary effect	−0.1642	0.255	−0.2171	−0.1155	42.47%

## 4 Discussion

As technology advances, the transformations ushered in by the information age have made mobile phones an essential component of everyday life (Zhang and Wu, 2020). However, addiction issues resulting from excessive mobile phone use are becoming increasingly prevalent, underscoring the importance of exploring mechanisms to enhance MPA management (Sahu et al., 2019). Previous studies have demonstrated that PA positively influences MPA, yet further research is needed to elucidate the precise mechanisms involved (Liu et al., 2019). Consequently, drawing upon both theoretical and empirical research, this study developed a chain mediation model aimed at examining the effects of PA among college students on MPA, as well as the mediating roles of self-control and resilience within this relationship. This endeavor seeks to expand upon previous research concerning the mechanisms linking PA to MPA and to enhance our understanding of the potential benefits of self-control and resilience. The present study identified three mediating pathways in the relationship between PA and MPA among Chinese university students, in which self-control and resilience to frustration had a significant effect on this relationship. Notably, PA impacts MPA through the sequential mediation of self-control and resilience. This provides new evidence that PA serves as a positive and effective health intervention, capable of enhancing self-control and resilience, thereby alleviating excessive mobile phone dependence and reducing MPA.

First, this study revealed a significant negative correlation between college students’ PA and MPA, a finding that aligns with prior research regarding the impact of PA on MPA (Li et al., 2023; Pirwani and Szabo, 2024). Thus, hypothesis H1 has been substantiated. The findings of this study further reinforced the connection between PA and MPA, indicating that active engagement in physical activities can diminish the sedentary behaviors of college students, consequently reducing the time spent on mobile phones and, thereby, decreasing the likelihood of MPA (Li et al., 2021). From the perspective of triadic theory, individuals, behaviors, and environments interact in a dynamic manner. PA, as an external stimulus, not only effectively enhances individuals’ physical health but also psychologically fortifies their resilience against stress, alleviates negative emotions, and thereby contributes to the reduction of addictive behaviors (Flynn et al., 2010). Research in neurophysiology has shown that PA can restore and regulate highly activated neuronal cells, thereby enhancing the adaptive capacity of mobile phone addicts to external changes (Guo et al., 2022). Furthermore, numerous animal studies suggest that neurotransmitters in the brain, such as dopamine, may mediate the relationship between PA and cognitive performance (Hou et al., 2024). Prolonged exercise fosters reward-related neuroplasticity in brain structures, potentially serving as a neural mechanism for enhancing self-control by diminishing an individual’s MPA in response to rewarding stimuli (Cassilhas et al., 2016). Empirical studies have demonstrated that PA can alleviate various issues associated with addiction symptoms, including withdrawal reactions, mood fluctuations, and reduced adaptive capacity (Chen et al., 2021). Therefore, considering the above studies and analyses, the present study concludes that increased levels of PA in university students can significantly enhance their resilience, which in turn reduces the incidence of MPA.

Secondly, this study reveals that college students’ PA appears to enhance self-control, subsequently diminishing their moderate PA. Thus, self-control mediates the relationship between college students’ PA and moderate PA. This finding aligns with prior research (Liu et al., 2024; Zhang et al., 2022), thereby validating hypothesis H2. The findings of this study further affirm the mediating role of self-control between PA and MPA. Through engaging in physical activity, college students enhance their self-control, thereby reducing the prevalence of negative behaviors associated with PA, among others (Zhong et al., 2021). Individuals with low self-control struggle to resist automatic, habitual behaviors, such as excessive mobile phone use, and are more likely to seek immediate gratification, succumbing to fleeting desires. When deprived of their mobile phones, they often feel disoriented and bored, a characteristic common among college students experiencing PA (Jiang and Zhao, 2016; Mei et al., 2018). Insufficient self-control leads to behavioral changes in college students in response to adverse emotions, consequently increasing the risk of MPA (Zhang et al., 2022). Thus, self-control can effectively inhibit unhealthy mobile phone usage. Prior research indicates that physical exercise can enhance inhibition and address control deficits in individuals experiencing MPA (Davis et al., 2011). When confronted with a cognitive task, those who engaged in regular exercise demonstrated significantly lower MPA scores (Zhou and Wang, 2022). These findings suggest that increased physical activity among college students may be advantageous in bolstering self-control, thereby preventing and mitigating MPA behaviors.

Thirdly, the findings indicate that resilience serves as an independent mediator in the relationship between PA and MPA among college students. In essence, college students may enhance their resilience through PA, thereby diminishing the prevalence of MPA (Ma et al., 2022a; Peng and Liu, 2010). This aligns with prior studies, thus validating Hypothesis 3. The results of this study further affirm the relationship between resilience and PA, demonstrating that sustaining a habit of PA enhances an individual’s adaptive capacity and stress tolerance, thereby augmenting resilience (Dunston et al., 2022; Jefferies et al., 2019). Moreover, the analysis of the interplay between resilience, PA, and MPA further highlights the critical role of resilience within this mechanism. This supports previous research indicating that PA can diminish the risk of MPA by fostering resilience and alleviating perceived stress (Zhao et al., 2022). Our findings lead us to hypothesize that a higher level of PA among college students correlates with greater resilience, enhancing their coping abilities and resilience to external challenges, ultimately reducing their likelihood of developing MPA (Ma et al., 2019).

Finally, this study established a chain mediation model linking PA and MPA through the mediating influences of self-control and resilience. The results indicate that self-control and resilience function as chain mediators in the relationship between PA and MPA, elucidating the mechanisms by which PA impacts MPA through the combined effects of these two factors. Our findings highlight a significant connection between self-control and resilience, corroborating previous research (Schuch et al., 2020). Based on this study’s results, we hypothesize that higher levels of PA among college students are associated with greater self-control, which subsequently enhances resilience, thereby mitigating negative emotions such as anxiety and improving emotional regulation, ultimately reducing the propensity for MPA.

## 5 Practical implications and limitations

In conclusion, the findings of this study substantiate the hypotheses posited in the research design. By integrating the variables of self-control and resilience, we have enriched the existing literature on the influence of PA on MPA and elucidated its underlying mechanisms. In alignment with the World Health Organization’s PA guidelines, we propose that the actual levels of PA may surpass the recommended thresholds. This assertion is grounded in the premise that PA yields significant mental benefits, alleviates negative emotions, and enhances physical well-being, among other advantages. Especially in the latter stages of the COVID-19 pandemic, physical dysfunction and mental distress, such as MPA, arising from confinement were, in part, attributable to diminished levels of physical activity (Wang et al., 2024). Our study’s findings indicate that university students ought to enhance their levels of PA, actively engage in exercise, and cultivate a consistent habit of physical engagement. This aligns with scholarly perspectives that view self-control as a muscle that can be strengthened (Liu et al., 2024). Such improvements can bolster psychological resilience, mitigate negative emotional states like anxiety and depression, and diminish maladaptive behaviors, including reliance on psychological interventions. Consequently, universities should not only encourage student participation in physical activities but also provide more opportunities for such engagement. For instance, they should monitor and guide students’ physical activity habits, address tendencies toward MPA, and regulate excessive mobile phone usage to foster their overall physical and mental well-being.

However, some limitations of this study should be mentioned when interpreting the findings. Firstly, the data collected in this study are cross-sectional data which can only explain the correlation between PA and MPA among university students but not the causal relationship. Further longitudinal or experimental studies are still needed in the future to explain and validate this causal relationship. Second, all subjects in this study were from one region in Sichuan Province, China, which limited the representativeness of the sample. Expanding the scope of the investigation to a national sample as well as a global sample is an inevitable trend for future research, in addition to adopting a more rigorous sample sampling method, which will further reduce bias. Third, from the perspective of data analysis, this study analyzed whether there were differences in PA, self-control, mental toughness and MPA by gender, but the findings showed no differences. Therefore, more research is needed to explain the relationship between gender differences in the above variables. Fourth, differences in types of exercise. This study only focused on the frequency of physical activity and did not delve into the different effects of different types of physical activity (e.g., team sports versus individual sports) on self-control and resilience. Fifth, the effect of individual differences. There may be differences in physical activity participation, self-control, and resilience among students of different genders, grades, and professional backgrounds, so it may not be possible to fully control the effects of these factors.

## 6 Conclusion

PA has been identified as a significant factor in diminishing the inclination toward MPA (Kim et al., 2015; Pereira et al., 2020). Self-control and psychological resilience function as intermediary chains between PA and MPA among college students. The findings of this study carry vital implications for college student populations, university institutions, and society at large in promoting PA to alleviate MPA.

First, to mitigate tendencies toward MPA, enhance self-control, and bolster resilience, it is essential for college students to engage in regular physical activity. Second, universities should prioritize the PA levels within the student body by providing accessible resources and facilities for physical engagement. Finally, society must recognize the prevalence of MPA, particularly among college students and younger populations, and work to foster motivation and positive perceptions of participation in physical activities, as well as improve the physical environment conducive to such engagement.

## Data Availability

The original contributions presented in the study are included in the article/Supplementary material, further inquiries can be directed to the corresponding author.
